# Temperature Shift Alters DNA Methylation and Histone Modification Patterns in Gonadal Aromatase (*cyp19a1*) Gene in Species with Temperature-Dependent Sex Determination

**DOI:** 10.1371/journal.pone.0167362

**Published:** 2016-11-30

**Authors:** Yuiko Matsumoto, Brette Hannigan, David Crews

**Affiliations:** 1 Department of Integrative Biology, University of Texas at Austin, Austin, Texas, United States of America; 2 Department of Pharmacy, University of Texas at Austin, Austin, Texas, United States of America; Massachusetts General Hospital, UNITED STATES

## Abstract

The environment surrounding the embryos has a profound impact on the developmental process and phenotypic outcomes of the organism. In species with temperature-dependent sex determination, gonadal sex is determined by the incubation temperature of the eggs. A mechanistic link between temperature and transcriptional regulation of developmental genes, however, remains elusive. In this study, we examine the changes in DNA methylation and histone modification patterns of the *aromatase* (*cyp19a1*) gene in embryonic gonads of red-eared slider turtles (*Trachemys scripta*) subjected to a temperature shift during development. Shifting embryos from a male-producing temperature (MPT) to a female-producing temperature (FPT) at the beginning of the temperature-sensitive period (TSP) resulted in an increase in *aromatase* mRNA expression while a shift from FPT to MPT resulted in decreased expression. DNA methylation levels at CpG sites in the promoter of the aromatase gene were high (70–90%) at the beginning of TSP, but decreased in embryos that were incubated at constant FPT and those shifted from MPT to the FPT. This decrease in methylation in the promoter inversely correlated with the expected increase in *aromatase* expression at the FPT. The active demethylation under the FPT was especially prominent at the CpG site upstream of the gonad-specific TATA box at the beginning of TSP and spread downstream of the gene including exon1 as the gonad development progressed. In embryos incubated at FPT, the promoter region was also labeled by canonical transcriptional activation markers, H3K4me3 and RNA polymerase II. A transcriptional repression marker, H3K27me3, was observed in temperature-shifted gonads of both temperature groups, but was not maintained throughout the development in either group. Our findings suggest that DNA hypomethylation and H3K4me3 modification at the *aromatase* promoter may be a primary mechanism that releases a transcriptional block of *aromatase* to initiate a cascade of ovarian differentiation.

## Introduction

Primordial gonads are initially bipotential; namely, having the capacity to develop into either testes or ovaries when they are generated from the coelomic surface of mesonephrous tissue during embryonic development. Differentiation of the embryonic gonads into one of the developmental trajectories (i.e., testes or ovaries) depends on either heritable genetic factors (i.e., genotypic sex determination, GSD) or the physical and biotic environment (i.e., environmental sex determination, ESD). Although gonad determination in mammals and birds is governed by the presence or absence of sex chromosomes, gonadal differentiation in non-mammalian vertebrates is susceptible to various environmental factors, especially temperature [1–3]. For instance, all crocodilians studied to date, many turtle, and some lizard species have shown little evidence of genetic differences between sexes; instead, temperature appears to be a sole determinant of gonad sex during embryonic development [1,4]. Recent studies reported a more complicated and yet lasting effects of temperature, which the organism experienced during development, on the physiology of the individual such as progeny's sex ratio and gonad determination system [5,6]. The gonadal sex of several fish species with sex chromosomes is also influenced by exposure to extreme temperatures, which produces a skewed sex ratio [3,7]. This form of temperature-driven ESD system is known as temperature-dependent sex determination or TSD. Comparative studies have shown that genes and regulatory pathways involved in sex differentiation are conserved across vertebrates although the hierarchy of gene regulatory pathways during upstream sex determination process seems to considerably vary. In a TSD system, the initial mechanism linking an upstream trigger, i.e., ambient temperatures, and the downstream modifiers involved in the regulation of relevant gene expression remain largely unknown.

Aromatase (*cyp19a1*) is an enzyme that irreversibly catalyzes androgens into estrogen in the steroidogenesis pathway. In mammals, the role of aromatase is essential to folliculogenesis and steroid hormone production in adult females but there is little evidence of aromatase involvement in gonad development and differentiation in the fetus [8–10]. In many non-mammalian vertebrates, however, aromatase appears to have a vital role in determining the fate of gonadal sex of the embryo by regulating local estrogen production in the gonads [7,11–13]. A recent study showed that overexpression of *aromatase* in genetically male chicken was sufficient to direct the embryonic gonads to differentiate into ovaries [14]. In species with TSD, the expression of *aromatase* in embryonic gonads is female-producing temperature (FPT) specific and the expression is almost undetectable at a male-producing temperature (MPT) [12,13,15]. In red-eared slider turtles (*Trachemys scripta*), for example, an dramatic increase of *aromatase* mRNA expression at FPT starts close to the middle of the temperature sensitive period (TSP), at stage 19, and reaches its peak at more than 10,000-fold increase relative to baseline (stage 16) right after TSP (stage 23) [16,17]. Upon hatching, the mRNA expression drops back down to levels similar to that observed around stage 19–21. These findings suggest that the activation of the *aromatase* may be a pivotal branching point in determining gonad sex trajectories. Despite the critical role of aromatase and subsequent estrogen production in non-mammalian sex determination, however, the mechanisms underlying the spatial (gonads vs. other tissues), temporal (early vs. late development), and quantitative (male vs. female) regulation of the aromatase gene remain mostly uncharacterized.

Accumulating evidence suggests that epigenetic modifications may be a missing link between the ambient temperature and the transcriptional regulation of *aromatase* during gonad determination. Epigenetic mechanisms, such as DNA methylation and histone modifications have been implicated as an important mediator in the process of phenotypic plasticity [18,19]. Recent studies suggest that temperature-specific expression of the *aromatase* during gonad determination is also directed by epigenetic mechanisms, especially DNA methylation. For example, in European sea bass (*Dicentrarchus labrax*), where gonadal sex is determined by genetic makeup but influenced extensively by temperature, high temperature-induced masculinization is accompanied by an increase in gonadal DNA methylation at the *aromatase* promoter and subsequent suppression of *aromatase* expression [20]. In red-eared slider turtles and another TSD species American alligator (*Alligator mississippiensis*), decreased *aromatase* promoter methylation and subsequent gene activation in the gonads are observed at FPT while the opposite is observed at MPT [21,22]. Although these studies suggest a relationship between the incubation temperature and DNA methylation levels during gonad determination, all available reports to date are limited to the correlational studies and whether the temperature is a causation of the change in DNA methylation has not been empirically examined.

Another critical epigenetic mechanism involved in the establishment of developmental gene expression patterns is histone modifications. Certain modifications to the histone tails, which in turn is recognized by different modifiers/proteins, are often investigated to predict the chromatin conformation that impacts the transcriptional accessibility of neighboring genomic regions [23,24]. Trimethylation of histone H3 at lysine 4 (H3K4me3) is one of the universal markers of an active promoter and is associated with euchromatin. H3k4me3 is also found in promoter regions that contain trimethylated histone H3 at lysine 27 (H3K27me3), which is a repressive histone modification; these promoters nonetheless are poised for activation upon receiving the proper developmental signals [25–27]. H3K4me3 modification to neighboring regions of a transcription start sites (TSS) often coincides with the localization of RNA polymerase II (RNAPII), of this localization is often inversely correlates with the local level of DNA methylation [23,28–30]. Although both DNA methylation and histone modifications are involved in affecting chromatin conformation, DNA methylation presumably elicits a long-term effect by stabilizing the repression of these genomic regions, while histone modifications involve only short-term, temporary changes in local chromatin structure [23]. Revealing how these epigenetic modifications are regulated by the ambient temperature and altered in response to sudden temperature changes at the *aromatase* will provide key insights into the mechanism underlying the developmental plasticity.

In the present study, we examined the changes in the epigenetic modifications of the *aromatase* and subsequent mRNA expression in embryonic gonads of red-eared slider turtles subjected to a temperature shift during development. Eggs from red-eared slider turtles initially incubated at either MPT or FPT regimens were shifted to the opposite temperature at the beginning of the TSP, embryonic stage 16. This shift has been shown to completely reverse the trajectory of gonadal differentiation, producing ovaries or testes matching its new environment [31]. The changes in *aromatase* mRNA expression, DNA methylation, histone modification, and RNAPII recruitment at the promoter in the gonadal tissue were quantitatively measured in subsequent developmental stages until hatching.

## Materials and Methods

This study was approved by IACUC protocol (#AUP-00149) at the University of Texas at Austin.

### Animals and study groups

**Fig 1:**
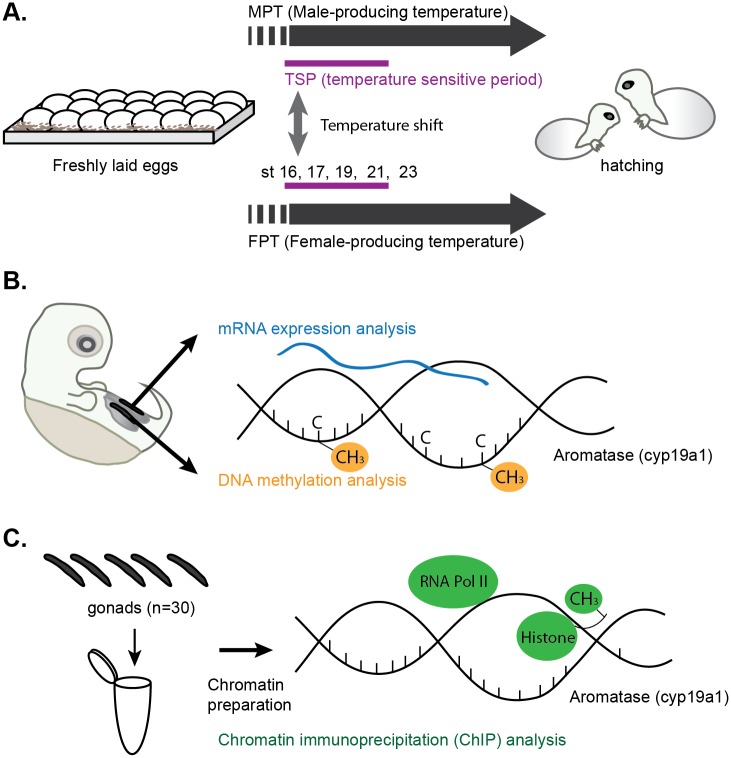
Experimental design. (A) Experimental design and stages of turtle development. Eggs were shifted to the opposite temperature regime at the beginning of the temperature sensitive period (TSP), embryonic stage (st) 16, to examine the effect of incubation temperatures on aromatase gene regulation. Corresponding embryonic stages for sample collections are indicated as st16, 17, 19, 21, and 23. (B) Experimental design for mRNA expression and DNA methylation analysis study of the aromatase gene. Gonads from embryos were divided for the two assays to analyze mRNA expression and DNA methylation, respectively. Data was also used for correlation studies. (C) Experimental design of the chromatin immunoprecipitation (ChIP) analysis. Embryonic gonads were pooled for chromatin preparation, and the enrichment of the protein of interest at the aromatase gene was examined by ChIP.

Freshly laid red-eared slider turtle eggs were purchased from Tangi turtle farm (Ponchatoula, LA, USA) during the months of May–July in 2014 and in 2015. Eggs obtained in the summer of 2014 were used for Chromatin immunoprecipitation (ChIP) experiments, and eggs obtained in 2015 were utilized for the RNA expression assay and DNA methylation assay. Eggs were harvested every day at the farm and shipped on the day of collection (day 1), ensuring that all eggs in the shipment were roughly at the same developmental stage. The gestational period of red-eared slider turtles is approximately 82 and 63 days at the MPT (26°C) and FPT (31°C) respectively. Eggs were maintained and processed in accordance with humane animal practices under approved IACUC protocol (#AUP-00149) at the University of Texas at Austin. Eggs were maintained at room temperature (RT) for the first 10 days upon arrival as the viability of the embryos could be assessed by candling. Those containing viable embryos were randomly assigned and incubated in incubators set at either MPT or FPT with moistened vermiculite until the beginning of TSP, Greenbaum’s embryonic stage 16 [32]. The average day to reach to the stage 16 is approximately 37 and 24 and days at MPT and FPT respectively. At stage 16, two cohorts of eggs were shifted from MPT to FPT (= m→FPT) or from FPT to MPT (= f→MPT) while the other cohorts served as control eggs maintained at MPT or FPT, respectively (Fig 1A).

### Sample collection

For the mRNA expression assay and DNA methylation assay, embryonic gonads were dissected at stages 16, 17, 19, 21, 23, and at hatching. Gonads collected at stage 16 served as baseline controls exposed to one temperature for the entirety of the study while the gonads collected at the other stages underwent either a temperature shift or remained at their respective original incubation temperatures. Each stage of the slider embryonic development takes approximately 3–4 days at MPT and 2 days at FPT [32,33]. For each embryo the gonadal tissue was dissected from the underlying adrenal-mesonephros tissue; the two gonads of each individual were separated to measure either mRNA expression or DNA methylation levels of the aromatase gene and the data examined for correlation studies (Fig 1B). Gonads for mRNA expression analysis were extracted in Trizol reagent (Thermo Fisher Scientific, Grand Island, NY), vortexed, and stored at -20°C until RNA isolation. Gonads for the DNA methylation assay were extracted, immediately snap-frozen in liquid nitrogen (LN_2_), and stored at -80°C until subsequent analysis. Ten embryos were collected for biological replicates and independently processed for both assays (n = 10 for both assays). One embryo in hatchlings from the m→FPT group failed to reverse the gonad sex (hatching with testes) and, therefore, was excluded from the study. To prepare chromatin for ChIP analysis, 15 embryos (a total of 30 gonads) were pooled into a single tube containing 500 μl cold PBS for each group at the following stages: 16, 19, 23, and at hatching. There was one baseline control (at a single constant temperature) at stage 16 while the other collection stages also included those groups that underwent a temperature shift. Upon harvesting, pooled samples were immediately processed for chromatin preparation (Fig 1C). Each group included three independent biological replicates of chromatin (i.e., 45 total embryos/group). In this cohort of eggs used for ChIP analysis, two embryos in the m→FPT group and three in the f→MPT group failed to reverse the gonad sex and, therefore, were excluded from the study.

### RNA isolation and analysis

Total RNA was isolated from a single gonad with Trizol reagent according to the manufacturer's protocol, dissolved in 10 μL nuclease-free water and treated using the Turbo DNA-free kit (Thermo Fisher Scientific, Waltham, MA). Due to a small amount of RNA, all RNA samples were subjected to reverse-transcription with the iScript cDNA synthesis kit (Bio-rad, Hercules, CA) without measuring the input RNA amount (20 μL of the final vol.). The complete removal of genomic DNA contamination was confirmed by the absence of PCR product in the no reverse transcriptase controls. Relative aromatase gene expression in 2 μL of cDNA was examined using the KAPA SYBR FAST qPCR kit (KAPA biosystems, Wilmington, MA) on the ABI ViiA7 real-time PCR system (Thermo Fisher Scientific). Aromatase primer sequences are shown in S1 Table. The summary of primer positions for all assays in the current study is presented along with CpG positions for DNA methylation assay in S1 Fig. Samples were run in triplicate, and the median value was used for analysis. All Ct values obtained by the primer set specific to the aromatase gene were normalized with the expression of the catalytic subunit of protein phosphatase 1 γ (PP1), which has previously been validated as an internal control for this species [13]. Primer specificity was monitored by melting curve analysis. Ct values were analyzed using the ΔCt method and represented as a fold change.

### Bisulfite-pyrosequencing determination of DNA methylation

Previously frozen embryonic gonads were thawed on ice, treated with bisulfite, and purified using the EZ DNA methylation-direct kit (Zymo research, Irvine, CA) according to the manufacturer's protocol. Bisulfite-treated gDNA was amplified using a nested PCR method with one set of outer PCR primers and two sets of inner primers to target 4 CpG sites within the previously identified aromatase core promoter and exon 1 (S1 Fig) [21]. PCR primer sequences are shown in S1 Table. The outer PCR primers were designed using the online software Bisulfite Primer Seeker by Zymo Research and the two inner sets of primers were designed by EpigenDx (Hopkinton, MA). Both of the reverse inner primers were labeled with biotin and HPLC purified. All primers were ordered from IDT (Integrated DNA Technologies, Coralville, IA). The first PCR was carried out using KAPA HiFi HotStart Uracil+ readyMix (KAPA) and the second using TAKARA EpiTaq HS (Clontech, Mountain View, CA). Conditions for the first outer PCR were as follows: 3 min at 95°C; 25 cycles of 20 sec at 98°C, 30 sec at 55°C, 30 sec at 68°C; and 3 min at 68°C. PCR products were then diluted 1:10 with nuclease-free water, and 1 μL of the dilution was used for the following inner PCR. Conditions for the secondary inner PCR were as follows: 50 cycles of 10 sec at 98°C, 30 sec at 52°C, 30 sec at 72°C. The specificity of the PCR reactions was confirmed in randomly selected samples by visualization on a 1.2% agarose gel. Biotin-labeled PCR products were sent to EpigenDx for quantitative pyrosequencing. Primers used for sequencing are shown in S1 Table. All submitted samples passed the quality assessment by EpigenDx. The raw data was analyzed and reported as a fraction of unconverted (methylated) cytosines at each CpG site of interest.

### Chromatin preparation

Freshly harvested gonad tissues in cold PBS were cross-linked by adding 37% formaldehyde to obtain a final working concentration of 1% and incubated at RT for 15 min. The cross-linking reaction was stopped by the addition of 2 M glycine, and the pooled samples were further incubated at RT for 5 min. Resulting tissues were washed three times with cold PBS containing 1 mM PMSF and 1% protease inhibitor and gently disaggregated using a dounce homogenizer into a single cell suspension. Cells were lysed with cell lysis buffer (5 mM PIPES, 85 mM KCl, 0.5% NP40) and incubated on ice for 15 min. After centrifugation, the cell pellets were lysed with nuclear lysis buffer (50 mM Tris-Cl pH 8.0, 10 mM EDTA, 1% SDS) and incubated on ice for 10 min. The lysates were sonicated 4 x 4 sec each with 4sec intervals at 60% power using a sonicator (Qsonica, LLC., Newtown, CT) on ice. After centrifugation at 14,000 rpm for 10 min at 4°C, the chromatin supernatants were aliquoted into 5 tubes (100 μL each), snap frozen in LN_2_, and stored at -80°C until the ChIP assay.

### Chromatin immunoprecipitation (ChIP) assay

Dynabeads Protein A (Thermo Fisher Scientific) was prepared by removing the original solution and resuspending them in the original volume with dilution buffer (0.01% SDS, 1% Triton X-100, 2 mM EDTA, 20 mM Tris-Cl, 150 mM NaCl) containing 1 mM PMSF. Previously-frozen chromatin was thawed on ice, suspended in three volumes of dilution buffer, and pre-cleared with the addition of 20 μL of prepared Dynabeads Protein A and gentle rotation for 2 hrs at 4°C. After separating the chromatin from the beads, they were incubated overnight at 4°C with 2–4 μg of one of the following antibodies: rabbit polyclonal H3K4me3 (Millipore, 07–473), rabbit polyclonal H3K27me3 (Millipore, 07–449), mouse monoclonal RNA Polymerase II CTD repeat (Abcam, ab817), or rabbit polyclonal anti-mouse IgG (Abcam, ab46540) as a negative control. These antibodies were previously shown to be compatible with ChIP or ChIP-seq experiments [34–37] and the amount of antibodies required was optimized earlier using chromatin collected from turtle embryonic gonads. Ten percent of the chromatin suspension was set aside to be diluted in elution buffer (100 mM NaHCO_3_, 1% SDS) and the remaining chromatin was used for the addition of rabbit IgG as a negative control. The antibody-protein complexes were bound to 50 μL Dynabeads by incubating at RT for 1 hr. The protein-beads complexes were then washed by gentle 15 min rotation at RT for each buffer listed in this order (number of washes, components): RIPA (x1, 50 mM Tris, 150 mM NaCl, 0.1% SDS, 0.5% sodium deoxycholate, 1% NP-40, 1 mM EDTA), High-salt (x1, 50 mM Tris, 500 mM NaCl, 0.1% SDS, 0.5% sodium deoxycholate, 1% NP-40, 1 mM EDTA), LiCl (x1, 50 mM Tris, 1 mM EDTA, 250 mM LiCl, 1% NP-40, 0.5% sodium deoxycholate), and TE buffer (x2). Antibody-protein complexes were eluted by gentle rotation with 500 μL elution buffer for 15 min at 65°C. This elution process was repeated twice (total final vol. of 1 mL). The chromatin samples and inputs were reverse cross-linked by incubating at 65°C overnight (< 15 hrs) with 5 M NaCl and 1 μg RNaseA (Thermo Fisher Scientific). The reaction was subsequently treated with buffer containing 20 μg Proteinase K, 5 mM EDTA, and 20 mM Tris-HCl for 1 hr at 45°C. DNA was purified with conventional phenol-chloroform methods and resuspended in 20 μL TE buffer.

### ChIP-qPCR

Relative enrichment of specific proteins was quantified using the KAPA SYBR FAST qPCR kit (KAPA biosystems) on the ABI ViiA7 real-time PCR System. Two μL of ChIP-isolated DNA was amplified using three sets of qPCR primers that recognize the following regions of the aromatase gene: A) a 5' upstream region including two putative heat shock factor (HSF) binding sites, B) a core promoter region including the TATA box and TSS, and C) a coding region within exon 1 (S1 Fig). Primer sequences are shown in S1 Table. Samples were run in triplicate and a median value was used for analysis. The primer specificity of the qPCR reaction was monitored by melting curve analysis. The obtained Ct values were normalized to the input so that samples and background noise (mock rabbit IgG) levels could be directly compared. For this purpose, a Ct value of the input was adjusted to 100% by subtracting a dilution factor of 10, namely log2 of 10. Next, a Ct value of the samples or mock rabbit IgG were subtracted from the adjusted input values and calculated as a percent of input (i.e., 100^2(normalized Ct)).

### Statistical analyses

All statistical analyses and graphic visualizations were carried out using R (The R project for Statistical Computing). The differences between the averages of the control (constant temperature) and the temperature-shifted groups for the qPCR data were analyzed with the Wilcoxon rank sum test. Differences in the averages of the pyrosequencing data within a stage and across the various stages at each CpG site were tested by the Kruskal-Wallis test by ranks, and by a post-hoc multiple comparison of a pairwise Wilcoxon rank sum test. Two variable measurements from the same individual—aromatase mRNA expression and percentage of DNA methylation at the aromatase gene—were assessed with the Spearman's rank correlation coefficient. The student t-test was performed for statistical differences in enrichment between the protein of interest and IgG background in the ChIP analysis. A *p*-value of less than 0.05 was considered to be statistically significant.

## Results

### A temperature shift alters the developmental expression of *aromatase* mRNA

**Fig 2:**
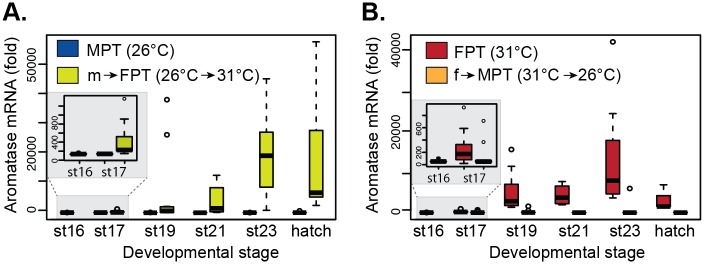
Gonadal aromatase mRNA expression during embryonic development. Slider eggs were incubated at MPT or shifted from MPT to FPT (A) and at FPT or shifted from FPT to MPT (B). Each time point includes data from 8–12 embryos. Data shown with a median value (thick black line), 25th and 75th percentiles (lower and upper boundary of box), and maximum and minimum values (whiskers). Outliers are indicated with open circles. All pairwise comparison between control and shifted temperatures within each stage showed statistical significances (p < 0.05 by Wilcoxon rank sum test, p values available in S2 Table). Stage 16 is represented as a single data point as it is a baseline assessment before the temperature-shift treatments. The small squares within the larger graphs are the magnified view of the results of stage 16 and 17. MPT = male-producing temperature, FPT = female-producing temperature. m→FPT = temperature shift from MPT to FPT. f→MPT = temperature shift from FPT to MPT. St = embryonic stage.

Quantitative PCR assessing gonadal aromatase mRNA levels showed a typical temperature-specific suppression and activation of the *aromatase* at MPT and FPT respectively throughout embryonic development (Fig 2A and 2B).

Temperature shifts at embryonic stage 16 resulted in an increase and decrease in expression for m→FPT (Fig 2A) and f→MPT (Fig 2B) gonads, respectively (S2 Table for *p*-values) as early as stage 17 (magnified graphs in panels in Fig 2A and 2B). This shift in the transcriptional trajectory continued throughout the remaining stages of development. When mRNA levels were compared between two control temperatures, MPT and FPT, the gonadal expression at stage 16 tended to be higher in the FPT group relative to those in the MPT group (P = 0.05, Wilcoxon rank sum; S2 Fig). Throughout the remaining stages, the difference in aromatase expression was significant between the two temperatures (S2 Fig). This sexually dimorphic expression of aromatase at the beginning of TSP occurred much earlier than what has previously been reported in differentiating gonads of other species (see Discussion).

### A temperature shift to a female-producing temperature is associated with promoter hypomethylation

**Fig 3:**
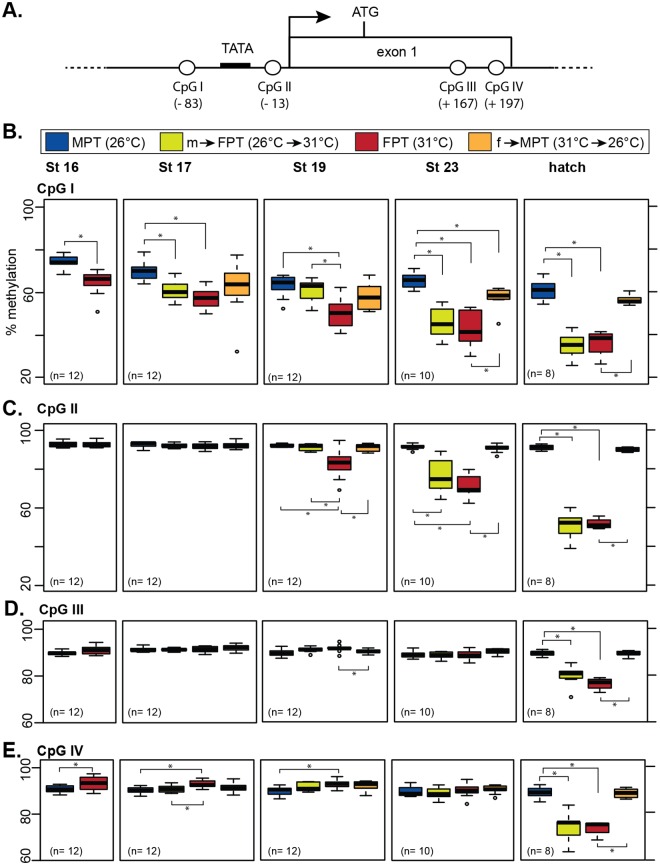
Developmental changes in DNA methylation patterns in the gonadal aromatase gene. (A) CpG dinucleotide positions (CpGI-IV) in the gonadal aromatase gene. TATA = putative TATA box. Black arrow = transcription start site. ATG = translation start site. Parenthesis indicates the base pair position of the CpG dinucleotide relative to the transcription start site counted as +1. (B) Percent DNA methylation of individual CpG sites in the gonadal aromatase gene in embryonic gonads. Data shown with a median value (thick black line), 25th and 75th percentiles (lower and upper boundary of box), and maximum and minimum values (whiskers). Outliers are indicated with open circles. Statistical differences between groups are marked with asterisks (p < 0.05, pairwise Wilcoxon rank sum test). Statistical results between the two temperature-shifted groups (m→FPT and f→MPT) within a stage and results across stages within a temperature group are not shown to simplify the graph (p-values available in S2 Table). The average number of individuals per group in each stage is indicated in parenthesis. MPT = male-producing temperature, FPT = female-producing temperature. m→FPT = temperature shift from MPT to FPT, f→MPT = temperature shift from FPT to MPT. St = embryonic stage.

We previously demonstrated that the level of DNA methylation at the aromatase promoter differs between the incubation temperatures [17,21]. However, the effect of a sudden temperature shift during TSP on DNA methylation has never been examined with quantitative methods and therefore the role of high vs. low incubation temperatures on gonadal determination has been inconclusive [21]. In the current study, we enhanced our DNA methylation analysis using a completely new batch of eggs with following changes; 1) using a quantitative method, i.e., pyrosequencing 2) examining four CpG dinucleotides located within a 300-bp region of the gonadal-specific transcription start site (TSS); two CpG sites (CpGI and II in the current study) that exhibited robust changes in methylation level in the previous study [21] and two additional CpG sites in exon1 (CpGIII and IV) (Fig 3A), 3) examining the extended developmental stages from stage 16, the earliest stage that embryonic gonads can be manually extracted, throughout development until hatching.

While screening this aromatase region, we also discovered a polymorphic CpG site in the 5'UTR located 38-bp downstream of the TSS (S1 Fig). This CpG site, which contains the G/A polymorphism that can cause partial loss of the CpG site in some individuals, was therefore excluded from our DNA methylation analysis. We found that the individual genotype of this polymorphic site had no correlation with the expression of aromatase mRNA in embryonic gonads.

The methylation level at CpGI, located approximately 50-bp upstream of the TATA box, was significantly lower in gonads developed at FPT than those developed at MPT; this pattern was already evident at stage 16, the beginning of TSP (Fig 3B). While the methylation level at CpGI in gonads at MPT remained relatively unchanged throughout development, it was increasingly hypomethylated in the FPT group as development progressed (Fig 3B). At this CpG site, a temperature shift from MPT to FPT at stage 16 resulted in not only a decrease in methylation at stage 17 but also a continual hypomethylation in the subsequent stages, which was the pattern similarly observed at FPT. A temperature shift from FPT to MPT also resulted in a rapid epigenetic response in embryonic gonads as exhibited by a high level of methylation similar to that observed at MPT (Fig 3B). The temperature-specific effect on DNA methylation at CpGI became more apparent as development progressed, with the most evident difference at hatching.

Despite being proximal to CpGI, methylation level at CpGII remained unchanged during early TSP, stage 16 and 17 (Fig 3C). DNA methylation pattern at CpGII did, however, eventually become similar to the one observed at CpGI, exhibiting a gradual decrease in the FPT phenotype groups (FPT and m→FPT) while remaining high in the MPT phenotype groups (MPT and f→MPT) from stage 19 till hatching (Fig 3C). DNA methylation levels at CpGIII and IV, both located within the first exon of the aromatase gene, did not appear to exhibit differences among the four temperature groups until hatching (Fig 3D and 3E). At CpGIV, the methylation level in the FPT group was even higher than those at MPT at stage 16, 17, and 19 (Fig 3E). At hatching, however, the methylation patterns at both CpGIII and IV were similar to the ones at the other CpG sites, i.e., hyper- and hypo- methylation in MPT and FPT phenotype groups respectively (Fig 3D and 3E).

### DNA methylation at the aromatase promoter is inversely correlated with mRNA expression

**Fig 4:**
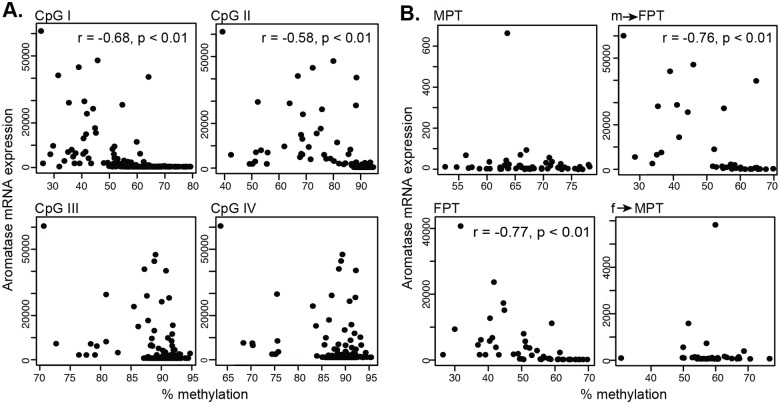
Scatter plots of aromatase mRNA expression against DNA methylation in gonads. (A) Data from embryos were plotted according to each CpG position. Correlation coefficient (r) and p-values were examined by Spearman's rank correlation coefficient. (B) Aromatase mRNA expression was plotted against DNA methylation at CpGI according to the different temperature groups. MPT = male-producing temperature, FPT = female-producing temperature. m→FPT = temperature shift from MPT to FPT, f→MPT = temperature shift from FPT to MPT.

Next, we examined if any correlation existed between mRNA expression and DNA methylation levels in each embryo. We found a significant but moderate negative relationship [38] at CpGI and CpGII sites (Fig 4A).

When this correlation was further divided by each temperature group, only CpGI in FPT phenotype groups showed a significant negative correlation between mRNA expression and DNA methylation (Fig 4B). This correlation was specific to those in the female temperature probably due to the inherent low level of aromatase mRNA expression in embryos at MPT, which is inadequate for this type of association study. When this link was examined by the various stages of development, DNA methylation at CpGI moderately correlated with mRNA expression at all stage though the correlation was observed at later stages for CpGII, III, and IV (S3 Fig).

### H3K4me3 modification and RNAPII recruitment to the promoter are hallmarks of ovarian differentiation

**Fig 5:**
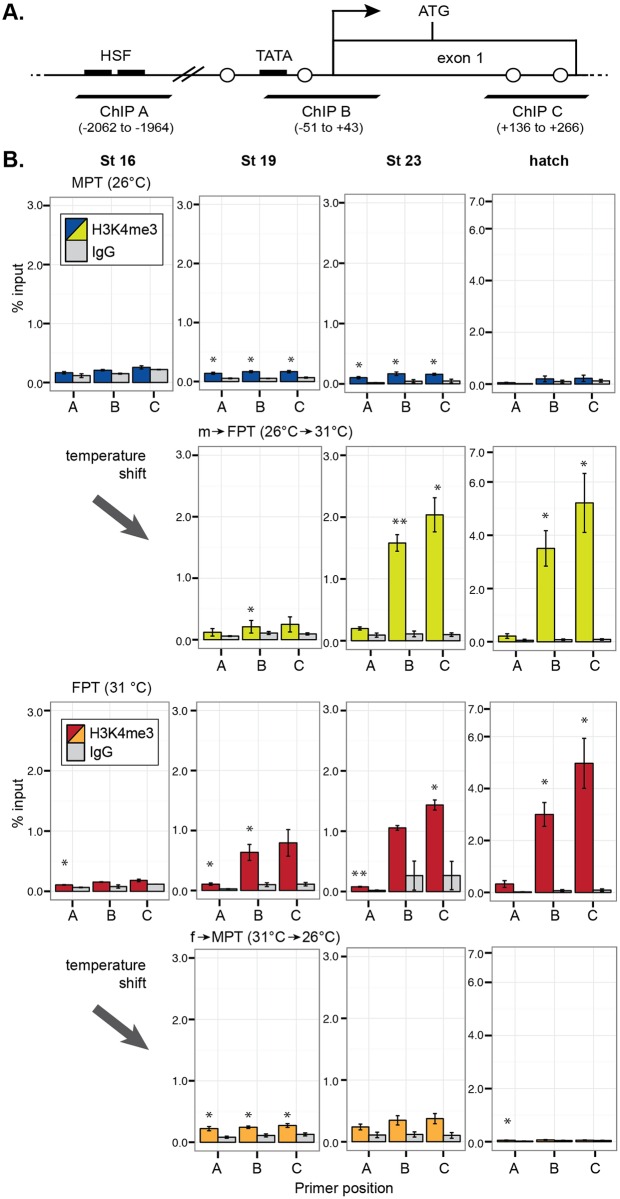
Enrichment of H3K4me3 at the gonadal aromatase gene during development. (A) qPCR primer (ChIP A-B) positions in the gonadal aromatase gene. TATA = putative TATA box. Black arrow = transcription start site. ATG = translation start site. Open circles = CpG dinucleotide positions from Fig 3. Parenthesis indicates the base pair position relative to the transcription start site counted as +1. (B) Quantitative enrichment of H3K4me3 detected by ChIP-qPCR. Each bar represents mean percentage of input ± s.e.m. in three biological (pooled gonads) replicates. Statistically significant changes between the protein of interest and rabbit IgG (IgG) negative control (background) are indicated with an asterisk (P < 0.05, student-t test). MPT = male-producing temperature, FPT = female-producing temperature. m→FPT = temperature shift from MPT to FPT, f→MPT = temperature shift from FPT to MPT.

We further examined developmental changes in histone modifications, namely the H3K4me3 and the recruitment of RNAPII as markers for active transcription and H3K27me3 for repressed transcription. Three sets of qPCR primers were used to detect the following regions of the aromatase gene: A) a 5' upstream region including two putative heat shock factor (HSF) binding sites, B) a core promoter region including the TATA box and TSS, and C) a coding region within exon 1 (Fig 5A).

In eggs incubated at MPT, H3K4me3 modification was not detected in the aromatase promoter region at embryonic stage 16 and at hatching though a moderate enrichment was observed at the middle of and after TSP (stage 19 and 23, respectively) (top panels in Fig 5B). In m→FPT embryos, the enrichment of H3K4me3 was manifested throughout development (second top panels in Fig 5B). A similar developmental increase in H3K4me3 was observed in the FPT group, which was particularly prominent at later stages (second bottom panels in Fig 5B). This FPT-specific H3K4me3 modification during stage 23 and at hatching were specific to the TSS and exon1 regions where an active transcription typically occurs, while it was less common at the region approximately 2kb upstream of the gene. In f→MPT embryos, H3K4me3 modification was observed across all investigated genomic regions during stage 19, but this enrichment ceased at later developmental stages—stage 23 and at hatching (bottom panels in Fig 5B).

**Fig 6:**
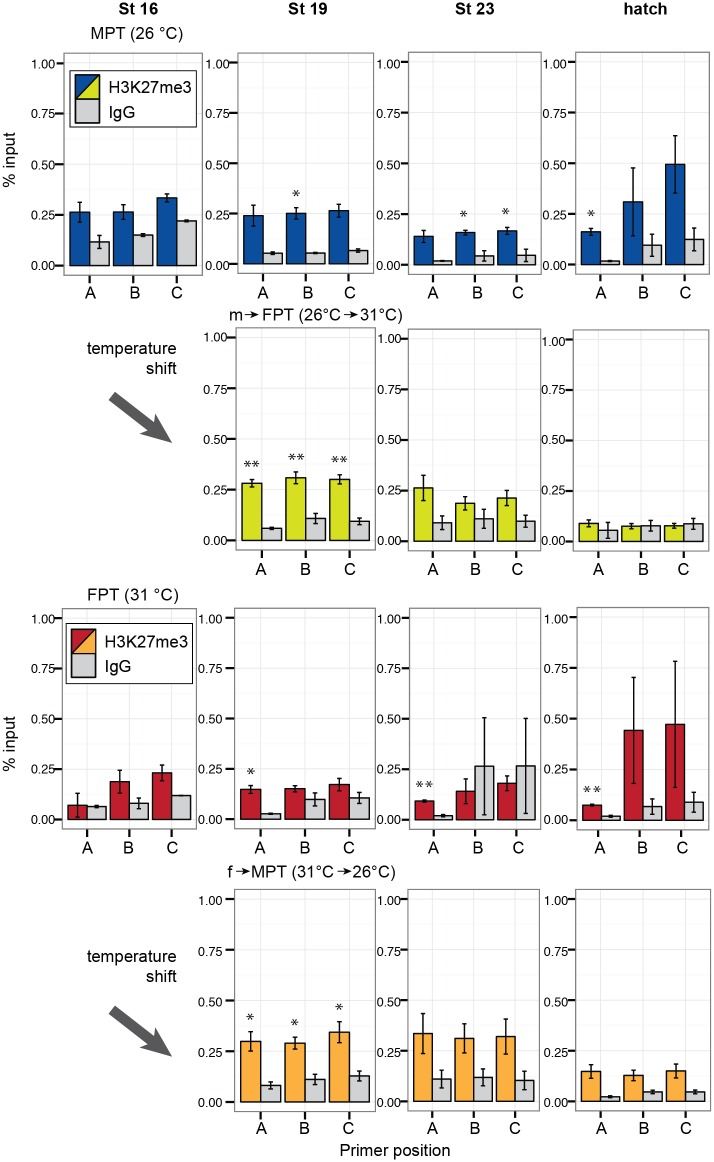
Enrichment of H3K27me3 at the gonadal aromatase gene during development. Quantitative enrichment of H3K27me3 detected by ChIP-qPCR. Each bar represents mean percentage of input ± s.e.m. in three biological (pooled gonads) replicates. Statistically significant changes between the protein of interest and rabbit IgG (IgG) negative control (background) are indicated with an asterisk (P < 0.05, student-t test). MPT = male-producing temperature, FPT = female-producing temperature. m→FPT = temperature shift from MPT to FPT, f→MPT = temperature shift from FPT to MPT.

Unlike H3K4me3, the repressive transcriptional marker H3K27me3 did not appear to have a specific pattern in any of the temperature groups. In the eggs incubated at MPT, H3K27me3 modification was not observed until stage 19 at when a slight enrichment of H3K27me3 was observed, but the degree of enrichment was not as obvious as the one observed with the H3K4me3 modification (top panels in Fig 6).

In m→FPT embryos, the H3K27me3 modification was evident across all investigated gene regions at stage 19; however, the modification disappeared during stage 23 and at hatching (second top panels in Fig 6). In eggs incubated at FPT, the H3K27me3 modification was not observed at stage 16 (second bottom panels in Fig 6). During stage 19 till hatching, the H3K27me3 modification in the FPT group was specific to the upstream region of the aromatase gene. In f→MPT embryos, a pattern similar to m→FPT embryos was observed for the H3K27me3 modification, namely, evident at stage 19, but gone during the late developmental stages (bottom panels in Fig 6).

**Fig 7:**
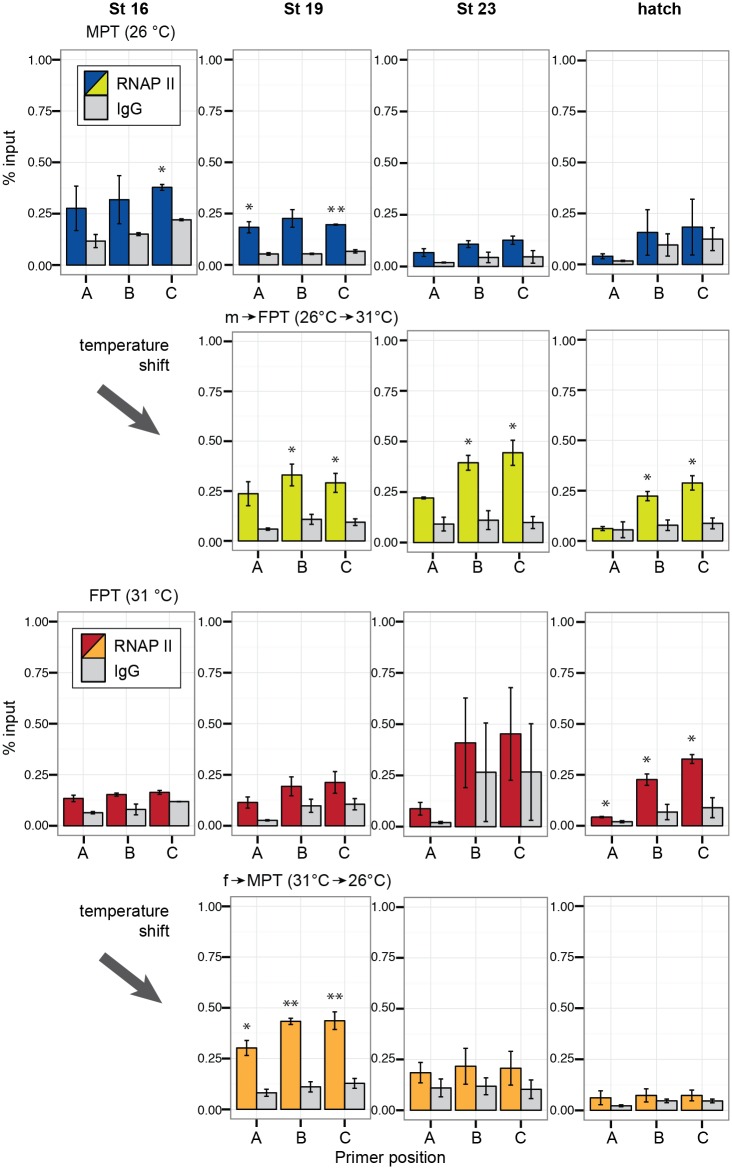
Enrichment of RNA polymerase II at the gonadal aromatase gene during development. Quantitative enrichment of RNA polymerase II detected by ChIP-qPCR. Each bar represents mean percentage of input ± s.e.m. in three biological (pooled gonads) replicates. Statistically significant changes between the protein of interest and rabbit IgG (IgG) negative control (background) are indicated with an asterisk (P < 0.05, student-t test). MPT = male-producing temperature, FPT = female-producing temperature. m→FPT = temperature shift from MPT to FPT, f→MPT = temperature shift from FPT to MPT.

RNAPII recruitment to the aromatase promoter was observed at the early to middle stages of TSP (stage 16 and 19) in embryos at MPT; however, no enrichment was observed at the late developmental stages as expected (top panels in Fig 7).

In m→FPT embryos, RNAPII recruitment was observed exclusively at the promoter and coding regions through the entire progress of development (second top panels in Fig 7), which coincided with the H3K4me3 enrichment. The RNAPII recruitment was only observed at hatching in eggs incubated at FPT (second bottom panels in Fig 7). In f→MPT embryos, RNAPII was observed only at stage 19 and not in the remaining developmental stages, which was the pattern similarly observed at MPT (bottom panels in Fig 7).

## Discussion

The expression of *aromatase* in differentiating gonads have been examined in various species, however, the actual role of aromatase during gonad sex determination is often regarded as a secondary effect; that is to facilitate the differentiation process that follows once the gonadal trajectory is fixed by the environmental cue. It is due to an absence of *aromatase* expression until midway of the gonad determination period as reported in species such as chicken [11], American alligator [12], and other turtle species [39,40]. We found however, that the baseline *aromatase* mRNA levels from stage 16 embryonic gonads already tended to significantly different between MPT and FPT (p = 0.05, Wilcoxon rank sum; S2 Fig). By stage 17, the difference in aromatase expression was significant between the two temperatures (S2 Fig). This sexually dimorphic expression of aromatase at the beginning of TSP was observed much earlier than what has previously been reported in differentiating gonads of other species. Consistent with this observation, the DNA methylation levels at the CpGI site, which is located approximately 50-bp upstream of the gonad-specific TATA box, significantly differed between these two incubation temperatures as early as stage 16 (Fig 3B). This suggests that the incubation temperature may have already exerted its effect and therefore predetermined the direction of aromatase transcription before the temperature shift at stage 16 at least in our model system. In support of this hypothesis, relative to the FPT control group, we discovered a remarkable variation in mRNA expression among different embryos in the m→FPT group regardless of the resulting uniform ovarian phenotypes among the cohort at hatching (Fig 2A and 2B). It suggests that incubation of eggs at MPT before stage 16 may predetermine the testicular gonadal development in differentiating gonads and therefore, affects the degree of *aromatase* expression in each embryo when a new environmental temperature is suddenly introduced. In red-eared slider turtles, the morphological/structural differences in gonads incubate at MPT and FPT is not apparent until the middle-end of TSP, at stage 19–20 [41]. The current study indicates that the temperature-specific transcriptional makeup and the subsequent transcription of *aromatase* are established much earlier in development than the morphological changes. It suggests that aromatase has a much more active role in determining the gonad sex trajectory than what is commonly perceived.

Our DNA methylation assay demonstrates that the relatively high levels of DNA methylation is already established throughout the aromatase promoter and exon1 regions at the beginning of TSP. The FPT seems to play an active role in the demethylation of aromatase promoter, especially targeting the upstream region of the gonad-specific TATA box (CpGI) at the beginning of TSP; this mode of action spreads downstream of the TATA box (CpGII) and exon1 (CpGIII and IV) as the gonad development progresses. In contrast, the role of MPT, which seems to be responsible for maintaining the high levels of methylation throughout development, seems rather passive. This asymmetric change of DNA methylation between two temperatures may indicate a critical mechanistic aspect of the regulation of *aromatase* gene during development.

The temperature shift at the beginning of TSP had an effect on the levels of DNA methylation at CpGI in the early stages, but not the other CpG sites. Instead, alterations to methylation at CpGII, located downstream of the TATA box, and CpGIII and IV, both located in the exon 1, were influenced by the temperature shift much later in development (Fig 3B–3E). It suggests that unlike CpGI, DNA methylation at these CpG sites may not be directly accountable for the initiation of the temperature-dimorphic expression of gonadal aromatase, which becomes apparent at stage 17. Rather, they may collectively be more involved in the stabilization of aromatase transcription in the differentiated ovaries.

Other studies in species with TSD demonstrate this temperature-specific change in DNA methylation in other, but not all, genes involved in the process of gonad determination. In the American alligator, a gene promoter of the actively transcribed testicular differentiation marker *Sox9* exhibits decreased methylation at MPT in differentiating gonads [22]. In European sea bass, temperature-directed methylation change to the aromatase promoter is observed only in the gonadal tissues but not brain and also specific exclusively to the aromatase gene [20]. We previously observed that a gene promoter of *FoxL2*, an ovarian differentiation marker, exhibited indistinguishable levels of DNA methylation in both MPT and FPT in the gonads of red-eared slider turtles during TSP, regardless of the observed temperature-dependent dimorphic mRNA expression of this gene (Y. Matsumoto, unpublished observations). Chemically-induced ovaries by exogenous estrogen treatment do not seem to follow the female-specific methylation pattern at the aromatase promoter even though the mRNA expression exhibits an expected increase by this treatment in species such as chicken [42], European sea bass [20] and red-eared slider turtles [17]. Taken together, these findings suggest that female-specific DNA methylation is one of the primary tools for priming the aromatase genome in response to both temperature and genetic cues, allowing the release of the transcriptional blockade to initiate a cascade resulting in the ovarian differentiation.

As predicted, the histone marker H3K4me3 enriches at the TSS and exon1 regions of the aromatase gene, which is where an active transcription typically occurs in the FPT phenotype groups (Fig 5B). The opposing marker H3K27me3 showed enrichment at stage19, the middle of TSP, in both shifted-temperature groups (Fig 6). In this study we did not find the typical H3K27me3 co-localization with H3K4me3 as has been observed in active or inactive, paused developmental genes [25–27]. Though we cannot completely rule out the possibility that this inconsistency was due to limitations to our experimental method (e.g. antibody efficiency), our study suggests that the silencing of the aromatase gene in the MPT phenotype groups may be mediated through other mechanisms outside of the repressive H3K27me3 mark, especially at the core promoter region.

H3K4me3 and DNA methylation is often in a mutually exclusive relationship [43,44]. We observed at the TATA promoter region that FPT-specific DNA demethylation occurs as early as stage 16 while H3K4me3 marks became prominent at the later stages of TSP (Figs 3B vs. 5B). It suggests that the FPT-specific initiation of aromatase expression at this region is directed by the hypomethylation of the promoter region while MPT-specific hypermethylation excludes H3K4me3 mark. At exon 1 region, however, H3K4me3 marks were observed in the FPT phenotype groups before FPT-specific DNA demethylation (Figs 3D and 3E vs. 5B), suggesting DNA methylation may not be responsible for expelling H3K4me3 in this region.

In the current study we used an antibody against an unmodified RNA polymerase II CTD, which mainly recognizes a paused RNAPII [45]. We initially expected to see this form of RNAPII in the embryonic gonads incubated at both MPT and FPT, especially during the early developmental stages when the aromatase promoter is thought to be poised for a timely transcription. Instead, the recruitment of RNAPII was observed in both groups of the temperature-shifted embryos at stage 19 (Fig 7). The RNAPII enrichment in the m→FPT group was continuously observed at the promoter and coding regions, which were also occupied with H3K4me3 throughout development. We do not have a clear understanding as to why RNAPII becomes enriched in the promoter specifically in the temperature-shifted groups, but we speculate that the phenomenon may be a part of the stress- or heat-induced response of RNAPII that is often reported in other species [46,47]. Again, we cannot rule out the limitation of ChIP-qPCR technique to detect the enrichment of certain protein. However, the localization of RNAPII was coordinated with H3K4me3 to some extend especially in embryos at FPT and shifted embryos from MPT to FPT throughout development.

In chicken, neither differential H3K4me3/H3K27me3 ratio nor RNAPII recruitment between female and male embryonic gonads (day 19) was detected at the gonad-specific aromatase TSS regardless of the sexually dimorphic mRNA expression at this stage [42]. They did, however, find H3K27me3 marks in both differentiating gonads and H3K4me3 marks with RNAPII recruitment more noticeably in female gonads at a genomic region approximately 1 kb upstream of the gonadal TSS. The authors did not rule out the possibility of the existence of another, more crucial, ovarian-specific TSS in this region. Many mechanisms of aromatase transcriptional regulation in non-mammalian vertebrates still remain to be characterized at a genetic and global chromatin structural levels in future studies; as our study also notes, it is one of the key pieces in understanding the mechanism of gonad sex determination of non-mammalian vertebrates. In summary, our current findings identify the critical role of epigenetic modifications in the regulation of this key gene involved in gonad differentiation of non-mammalian species and more broadly, the environmental impact on the developmental process and phenotypic outcomes.

## Supporting Information

S1 Fig A combined map of primer positions for mRNA and ChIP analyses and the CpG dinucleotide positions (CpGI-IV) examined for DNA methylation in the gonadal aromatase gene. HSF = putative binding site for heat shock factors. TATA = TATA box. Black arrow = transcription start site. ATG = translation start site. Parenthesis indicates the base pair position relative to the transcription start site counted as +1. CpG pol = G/A polymorphism resulting in loss of a CpG site in some individuals (see Results section).
(TIF)

S2 Fig Gonadal aromatase mRNA expression during embryonic development (MPT and FPT groups only). Each time point includes data from 8–12 embryos. Data shown with a median value (thick black line), 25th and 75th percentiles (lower and upper boundary of box), and maximum and minimum values (whiskers). Outliers are indicated with open circles. Asterisks indicate statistically significant difference between MPT and FPT groups within a stage (*p < 0.05, ** p < 0.01 by Wilcoxon rank sum test). Small squares within the larger graphs are the magnified view of the results from stage 16 and 17. MPT = male-producing temperature, FPT = female-producing temperature. St = embryonic stage.
(TIF)

S3 Fig Scatter plots of aromatase mRNA expression against DNA methylation examined by embryonic stages. Correlation coefficient (r) and p-values were examined by Spearman's rank correlation coefficient. A plot without an r-value was not statistically significant.
(TIF)

S1 Table Primer sequences for mRNA expression analysis (A), pyrosequencing analysis (B), and ChIP analysis (C). (DOCX)

S2 Table P-values from statistical analyses of mRNA expression analysis (A) and pyrosequence analysis (B, C). (DOCX)
