# Supplementary figures and images for: Temperature Shift Alters DNA Methylation and Histone Modification Patterns in Gonadal Aromatase (cyp19a1) Gene in Species with Temperature-Dependent Sex Determination
Source: PLoS One. 2016 Nov 30;11(11):e0167362. doi: 10.1371/journal.pone.0167362 (PMC5130277; doi:10.1371/journal.pone.0167362)

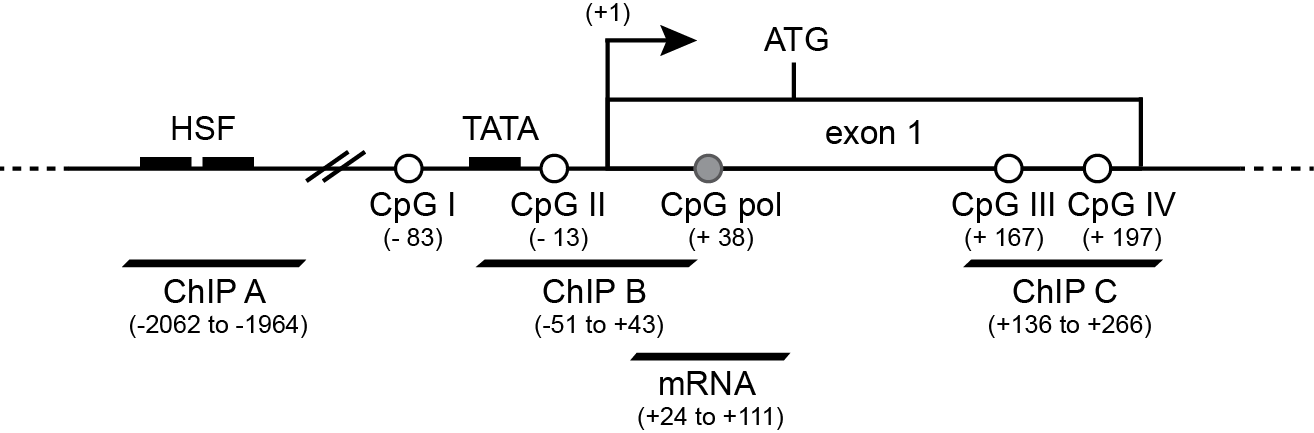

Supplement: S1 Fig — HSF = putative binding site for heat shock factors. TATA = TATA box. Black arrow = transcription start site. ATG = translation start site. Parenthesis indicates the base pair position relative to the transcription start site counted as +1. CpG pol = G/A polymorphism resulting in loss of a CpG site in some individuals (see Results section). (TIF) [file pone.0167362.s001.tif]

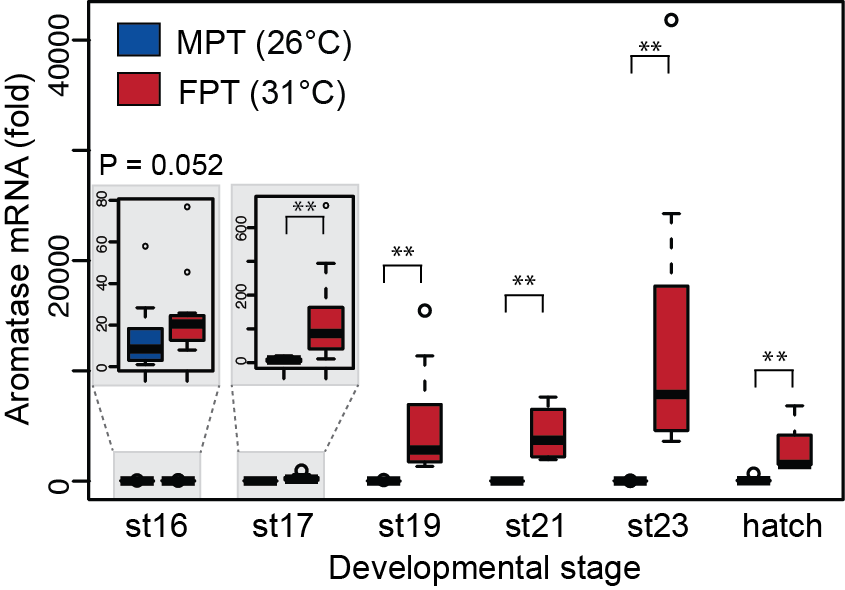

Supplement: S2 Fig — Each time point includes data from 8–12 embryos. Data shown with a median value (thick black line), 25th and 75th percentiles (lower and upper boundary of box), and maximum and minimum values (whiskers). Outliers are indicated with open circles. Asterisks indicate statistically significant difference between MPT and FPT groups within a stage (*p < 0.05, ** p < 0.01 by Wilcoxon rank sum test). Small squares within the larger graphs are the magnified view of the results from stage 16 and 17. MPT = male-producing temperature, FPT = female-producing temperature. St = embryonic stage. (TIF) [file pone.0167362.s002.tif]

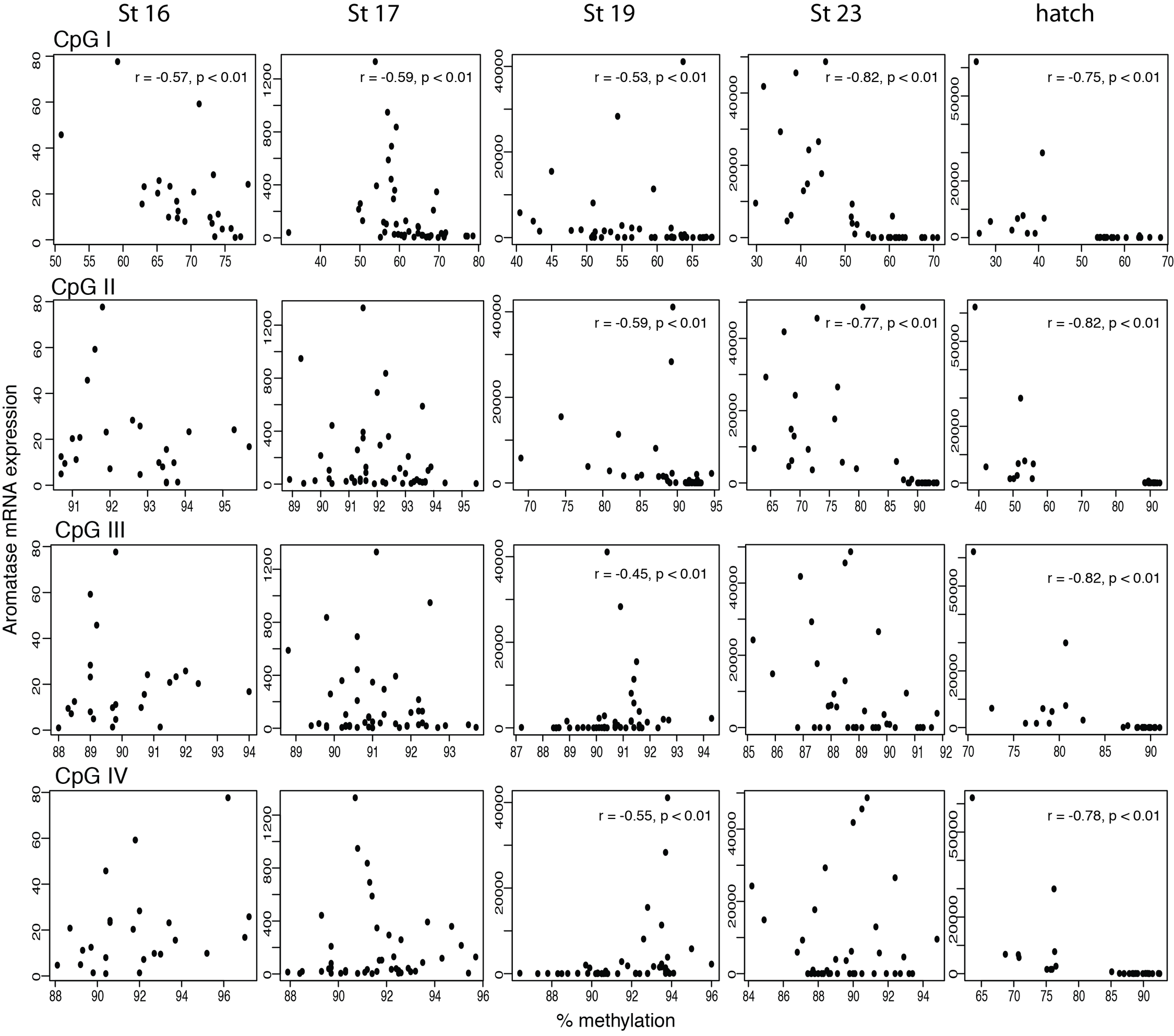

Supplement: S3 Fig — Correlation coefficient (r) and p-values were examined by Spearman's rank correlation coefficient. A plot without an r-value was not statistically significant. (TIF) [file pone.0167362.s003.tif]
