# Supplementary material for: Temperature Shift Alters DNA Methylation and Histone Modification Patterns in Gonadal Aromatase (cyp19a1) Gene in Species with Temperature-Dependent Sex Determination
Source: PLoS One. 2016 Nov 30;11(11):e0167362. doi: 10.1371/journal.pone.0167362 (PMC5130277; doi:10.1371/journal.pone.0167362)
Supplement: S1 Table — (DOCX) [file pone.0167362.s004.docx]

**Table S1.**

**A. Real-time qPCR primers for aromatase mRNA expression analysis**

| **Sequence (5' - 3')** | **Product size (bp)** |
| --- | --- |
| F- GGGTTGCAAGGTACCGATTA  R- ACAGTCTACTTTGCCTTTAGGG | 89 |

**F** = forward, **R** = reverse.

**B. PCR and sequencing primers for pyrosequence analysis**

| **Primer name** | **Target CpG** | **Sequence (5' - 3')** | **Product size (bp)** |
| --- | --- | --- | --- |
| Outer PCR primer | CpG I - IV | F- TTTTATATGTGTTGAGAGTTTAATTTTTTT  R- CATATTCCTTCTATATTTCCTAAATATACA | 668 |
| Inner PCR1 | CpG I & II | F- TGTTATTTTTGGGTAAAAAGTAAAT  R- bio-TTCAAAATACTAAAACACCTTTAATC | 180 |
| Inner sequence 1a | CpG I | F- TTTTGGGTAAAAAGTAAATT | - |
| Inner sequence 1b | CpG II | F- AGTAGTTAGTTTTATTATAAAAT | - |
| Inner PCR2 | CpG III & IV | F- GATATTGGAAATTTTGAATTTGAT  R- bio- CCTAATATTAATATTTCTTCATAATTCCA | 141 |
| Inner sequence 2 | CpG III & IV | F- GGAAATTTTGAATTTGATGTAT | - |

**F** = forward, **R** = reverse, **bio** = 5' biotin labeled.

**C. Real-time qPCR primers for ChIP analysis.**

| **Primer name** | **Sequence (5' - 3')** | **Product size (bp)** |
| --- | --- | --- |
| ChIP primer A | F- AAACACTATGGCAACACTTGA  R- GATGAGCTTACAGTCTAAGTAGAAGA | 101 |
| ChIP primer B | F- GTCTAAGCAGCCAGTCTCATTAT  R- TAATCGGTACCTTGCAACCC | 94 |
| ChIP primer C | F- CCCTGAATCTGATGCATTCC  R- ACCTGGTATTGATGTTTCTTCA | 131 |

**F** = forward, **R** = reverse.
