# Supplementary material for: Temperature Shift Alters DNA Methylation and Histone Modification Patterns in Gonadal Aromatase (cyp19a1) Gene in Species with Temperature-Dependent Sex Determination
Source: PLoS One. 2016 Nov 30;11(11):e0167362. doi: 10.1371/journal.pone.0167362 (PMC5130277; doi:10.1371/journal.pone.0167362)
Supplement: S2 Table — (DOCX) [file pone.0167362.s005.docx]

**Table S2.**

**A. *p*-values (data with P < 0.05 only, Wilcoxon rank sum) from statistical analysis of aromatase mRNA expression data (Fig. 2)**

| **pairwise (x)** | **pairwise (y)** | **stage (st)** | **p value** |
| --- | --- | --- | --- |
| MPT | m→FPT | st 17 | 1.134e-05 |
| MPT | m→FPT | st 19 | 2.219e-05 |
| MPT | m→FPT | st 21 | 1.588e-05 |
| MPT | m→FPT | st 23 | 3.093e-06 |
| MPT | m→FPT | hatch | 0.0003996 |
| FPT | f→MPT | st 17 | 0.004513 |
| FPT | f→MPT | st 19 | 5.917e-06 |
| FPT | f→MPT | st 21 | 2.165e-05 |
| FPT | f→MPT | st 23 | 0.0005485 |
| FPT | f→MPT | hatch | 0.006484 |

MPT = male-producing temperature, FPT = female-producing temperature, m→FPT = a temperature shift from MPT to FPT, f→MPT = a temperature shift from FPT to MPT.

**B. *p*-values (data with P < 0.05 only, pairwise Wilcoxon rank sum) from statistical analysis of pyrosequencing data of CpG sites (Fig. 3) within stages between m**→**FPT and f**→**MPT groups**

| **CpG position** | **stage (st)** | **pairwise (x)** | **pairwise (y)** | **p value** |
| --- | --- | --- | --- | --- |
| CpGI | st 23 | m→FPT | f→MPT | 0.004843548 |
|  | hatch | m→FPT | f→MPT | 0.001864802 |
| CpGII | st 23 | m→FPT | f→MPT | 0.005257917 |
|  | hatch | m→FPT | f→MPT | 0.007228904 |
| CpGIII | hatch | m→FPT | f→MPT | 0.007157952 |
| CpGIV | hatch | m→FPT | f→MPT | 0.007228904 |

MPT = male-producing temperature, FPT = female-producing temperature, m→FPT = a temperature shift from MPT to FPT, f→MPT = a temperature shift from FPT to MPT.

**C. *p*-values (data with P < 0.05 only, pairwise Wilcoxon rank sum) from statistical analysis of pyrosequencing data of CpG sites (Figure 3) within temperature groups across different stages**

| **CpG position** | **temperature** | **pairwise (x)** | **pairwise (y)** | **p value** |
| --- | --- | --- | --- | --- |
| CpGI | MPT | st16 | st19 | 0.0003644203 |
|  |  | st16 | st23 | 0.0005365338 |
|  |  | st16 | hatch | 0.0009128102 |
|  |  | st17 | st19 | 0.0252120778 |
|  |  | st17 | hatch | 0.0083281980 |
|  | FPT | st16 | st17 | 0.002598962 |
|  |  | st16 | st19 | 0.001972408 |
|  |  | st16 | st23 | 0.0002165018 |
|  |  | st16 | hatch | 0.006593407 |
|  |  | st17 | st23 | 0.0024000767 |
|  |  | st17 | hatch | 0.006593407 |
|  |  | st19 | hatch | 0.008791209 |
|  | m→FPT | st17 | st23 | 0.002671284 |
|  |  | st17 | hatch | 0.002671284 |
|  |  | st19 | st23 | 0.002671284 |
|  |  | st19 | hatch | 0.002671284 |
|  |  | st23 | hatch | 0.018648019 |
| CpGII | FPT | st16 | st19 | 0.001153780 |
|  |  | st16 | st23 | 3.092882e-05 |
|  |  | st16 | hatch | 0.005846032 |
|  |  | st17 | st19 | 0.001408203 |
|  |  | st17 | st23 | 3.092882e-05 |
|  |  | st17 | hatch | 0.005846032 |
|  |  | st19 | st23 | 5.846032e-03 |
|  |  | st19 | hatch | 0.005846032 |
|  |  | st23 | hatch | 0.005846032 |
|  | m→FPT | st17 | st23 | 0.001477764 |
|  |  | st17 | hatch | 0.001554002 |
|  |  | st19 | st23 | 0.001554002 |
|  |  | st19 | hatch | 0.001554002 |
|  |  | st23 | hatch | 0.001554002 |
|  | f→MPT | st17 | hatch | 0.02264419 |
| CpGIII | MPT | st16 | st19 | 0.018136641 |
|  |  | st17 | st23 | 0.004454528 |
|  | FPT | st16 | hatch | 0.02962413 |
|  |  | st17 | st23 | 0.02957668 |
|  |  | st17 | hatch | 0.02962413 |
|  |  | st19 | st23 | 0.02660622 |
|  |  | st19 | hatch | 0.02962413 |
|  |  | st23 | hatch | 0.01998002 |
|  | m→FPT | st17 | st23 | 0.002618541 |
|  |  | st17 | hatch | 0.002618541 |
|  |  | st19 | st23 | 0.002700338 |
|  |  | st19 | hatch | 0.002618541 |
|  |  | st23 | hatch | 0.002891561 |
|  | f→MPT | st17 | st19 | 0.02312196 |
|  |  | st17 | hatch | 0.006224462 |
| CpGIV | FPT | st16 | hatch | 0.03042197 |
|  |  | st17 | st23 | 0.04999859 |
|  |  | st17 | hatch | 0.01098901 |
|  |  | st19 | st23 | 0.04999859 |
|  |  | st19 | hatch | 0.01098901 |
|  |  | st23 | hatch | 0.01598402 |
|  | m→FPT | st17 | hatch | 0.002185093 |
|  |  | st19 | hatch | 0.002185093 |
|  |  | st23 | hatch | 0.001864802 |
|  | f→MPT | st19 | hatch | 0.04105938 |

MPT = male-producing temperature, FPT = female-producing temperature, m→FPT = a temperature shift from MPT to FPT, f→MPT = a temperature shift from FPT to MPT.
